# Left ventricular myocardial perfusion in young patients evaluated for hypertrophic cardiomyopathy at rest and during adenosine hyperemia using cardiac magnetic resonance imaging

**DOI:** 10.1186/1532-429X-16-S1-P163

**Published:** 2014-01-16

**Authors:** Tom Gyllenhammar, Eva Fernlund, Robert Jablonowski, Jonas Jögi, Henrik Engblom, Petru Liuba, Håkan Arheden, Marcus Carlsson

**Affiliations:** 1 Dept. of Clinical Physiology, Lund University, Lund University Hospital, Lund, Sweden; 2 Dept. of Pediatric Cardiology, Lund University, Lund University Hospital, Lund, Sweden

## Background

We aimed to determine if myocardial perfusion (MP) is decreased in young patients with hypertrophic cardiomyopathy (HCM) and first grade relatives without hypertrophy (subjects at risk) during adenosine induced myocardial hyperemia. Furthermore, we wanted to investigate if such a decrease was associated with diastolic dysfunction.

## Methods

Twelve controls (22.8 ± 4.5 years), fourteen subjects at risk (18.9 ± 3.8) and ten HCM patients (22.3 ± 6.4) were examined using echocardiography and cardiovascular magnetic resonance (CMR) at rest and during hyperemia (adenosine 140 μg/kg/min). Patients with left ventricular outflow tract obstruction were excluded from the study. Myocardial perfusion was calculated as the ratio of coronary sinus flow and left ventricular mass (LVM) from CMR. Myocardial fibrosis was assessed using late gadolinium enhancement. Diastolic function was quantified using both echocardiography and CMR. The Mann-Whitney test was used to compare the groups and results were considered significant if p < 0.05. Results are presented as mean ± SEM if not otherwise stated. The study was approved by the Ethical Board and informed consent was obtained.

## Results

There was no significant difference in MP (ml/min/g) at rest in controls, subjects at risk and HCM patients (0.8 ± 0.1, 1.0 ± 0.1, 0.9 ± 0.1, respectively, p = ns). During hyperemia, MP was similar in controls and subjects at risk (3.9 ± 0.3 and 5.0 ± 0.5, p = 0.11, but lower in HCM patients (2.5 ± 0.4, p < 0.05 compared to both). Even when fibrosis was excluded from LVM the MP was lower in HCM patients compared to the other groups. Two subjects showed mild diastolic dysfunction (E/e' = 16), otherwise none of the investigated subjects showed marked diastolic dysfunction (E/e'<15).

## Conclusions

Young HCM patients show decreased MP during hyperemia in non-fibrotic myocardium compared to controls even without diastolic dysfunction. These findings indicate that microvascular disease may be the cause of decreased MP in young patients with HCM. Funding. The Heart and Lung foundation, Region of Skane, Lund university

## Funding

The Heart and Lung foundation, Region of Skane, Lund University.

**Figure 1:**
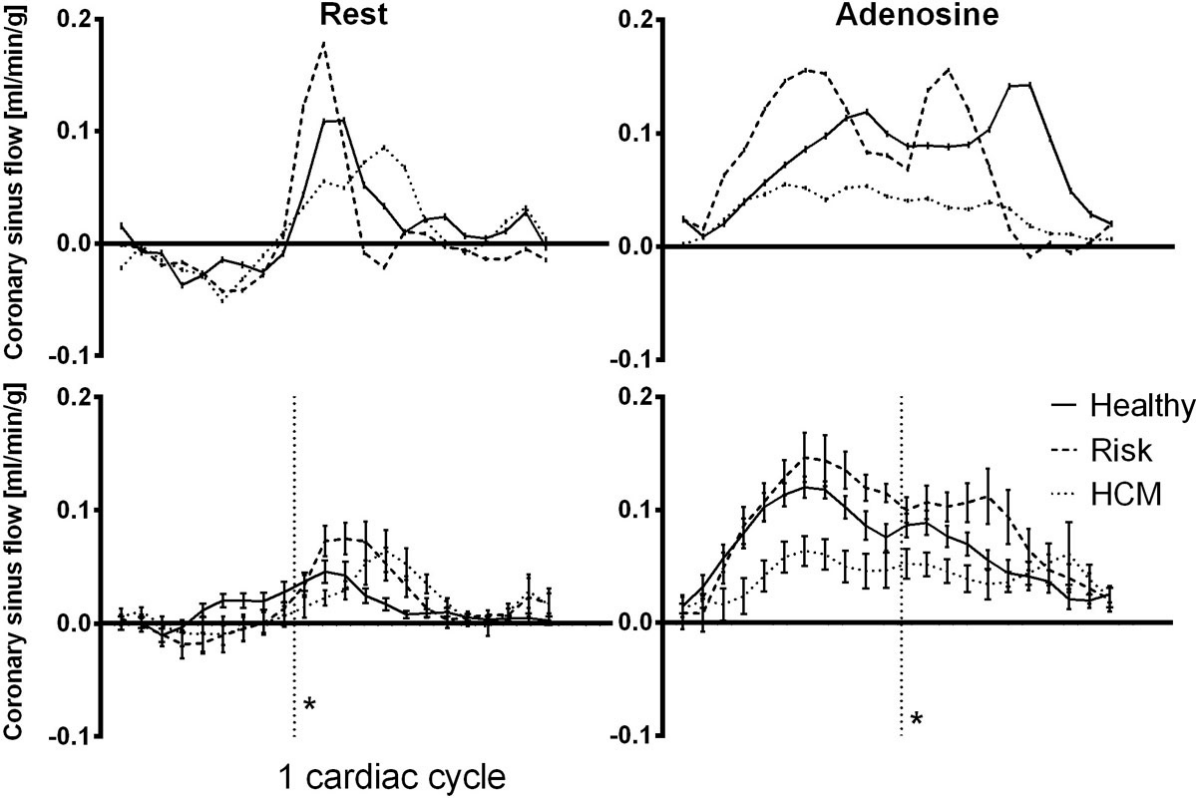
**Top panels: Typical coronary sinus flow (CSF) curves at rest (left) and adenosine hyperemia (right) during one cardiac cycle for one healthy subject (solid line), one subject at risk (broken line) and in one HCM patient (dashed line)**. Bottom panels: The CSF (mean ± SEM) for each group at rest (left panel) and adenosine hyperemia (right panel). Note that the CSF during adenosine hyperemia is lower throughout the cardiac cycle in HCM patients compared to controls and subjects at risk. * = average time of end systole.

**Figure 2:**
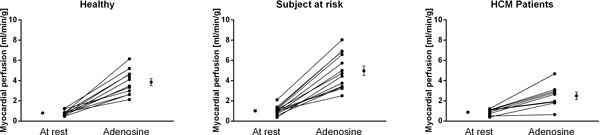
**Myocardial perfusion (MP) at rest and during adenosine hyperemia for each subject in all three groups**. Error bars show the mean ± SEM. During adenosine the MP was significantly lower in HCM patients compared to both controls and subjects at risk.

